# Randomised controlled trial of a psychotherapeutic intervention to improve quality of life and other outcomes in people who repeatedly self-harm: FReSH START study protocol

**DOI:** 10.1186/s13063-024-08369-2

**Published:** 2024-08-26

**Authors:** K. Farley, B. Copsey, A. Wright-Hughes, A. Farrin, C. Bojke, D. McMillan, C. D. Graham, R. Mattock, C. A. Brennan, C. Gates, A. Martin, A. Dowse, J. Horrocks, A. O. House, E. A. Guthrie

**Affiliations:** 1 UK Centre for Ecology & Hydrology, UK Centre for Ecology & Hydrology, Lancaster, UK; 2 University of Leeds Clinical Trials Research Unit, Leeds, UK; 3 University of Leeds Leeds Institute of Health Sciences, Leeds, UK; 4 Department of Health Sciences, University of York, Heslington, York UK; 5 University of Strathclyde, Glasgow, Scotland, UK; 6 Leeds Beckett University - City Campus: Leeds Beckett University, Leeds, UK; 7 Bionical Emas, Mercia Marina, Willington, UK

**Keywords:** Self-harm, Suicide, Talking therapies, Mental health

## Abstract

**Background:**

Self-harm is a major public health challenge, and repeated self-harm is common in those attending hospital following an episode. Evidence suggests psychological interventions could help people who self-harm, but few definitive studies have assessed their clinical and cost-effectiveness. Repeated self-harm is associated with poor quality of life, depression, suicide and increased health service costs which justify the development of psychotherapeutic interventions tailored for people with repeated self-harm.

**Methods:**

FReSH START is a multicentre individually 1:1 randomised controlled trial evaluating the clinical and cost-effectiveness of standard care plus psychological therapy or standard care alone for adults (≥ 18 years) presenting at an emergency department (ED) with repeated self-harm. Recruiting 630 participants, it includes an internal pilot, economic evaluation and process evaluation. The intervention will be delivered by mental health staff working in acute settings, with experience of assessing and managing risk in people presenting to emergency services with self-harm. Staff will be trained and supervised to deliver one of three specially adapted therapies: psychodynamic interpersonal therapy, cognitive behavioural therapy or acceptance and commitment therapy. Participants allocated to the intervention will receive one of the adapted therapies according to therapist allocation for up to 6 months via 12 weekly, one to one, 45–50-min sessions. The primary outcome is quality of life measured by the Clinical Outcomes in Routine Evaluation Outcome Measure at 12 months post-randomisation. Secondary outcomes include suicidal intent, depression and cost-effectiveness. Data are collected using hospital attendance records and online/postal/telephone questionnaires at 6 and 12 months post-randomisation, with resource use additionally collected at 3 and 9 months.

**Discussion:**

This protocol outlines a randomised controlled trial to investigate whether modified therapies are cost-effective and improve quality of life for people who repeatedly self-harm. Few interventions are proven to be deliverable in the NHS for this population. This study is strengthened by the involvement of qualified mental health workers experienced in managing risk as therapists.

**Trial registration:**

Registered on August 03, 2021.

IRAS number: 297939.

ISRCTN: 10.1186/ISRCTN73357210.

REC reference: 21/EE/0145.

Sponsor: University of Leeds.

**Supplementary Information:**

The online version contains supplementary material available at 10.1186/s13063-024-08369-2.

## Introduction

### Background and rationale {6a}

Self-harm is a major public health challenge with estimated lifetime prevalence of 5–6% [1] and the reason for 220,000 hospital attendances annually in England and Wales [2]. Repetition of self-harm is common with 70% of those attending hospital following an episode of self-harm reporting previous episodes and about 25% who will then attend for a subsequent act during 18-month follow-up [3]. Up to 20% of those who present to hospital report a history of over five acts [3]. For those seen in hospital after an episode which represents at least their third attendance, more than 50% will go on to a further repeat attendance [2]. It is assumed that 40,000–50,000 hospital attendances a year are accounted for by those who repeatedly self-harm [4]. Most is known about hospital attendance because of ease of data collection, but as many episodes again do not lead to hospital attendance [4, 5].

Repeated self-harm is associated with depression and misuse of alcohol, poor quality of life and problems with interpersonal and social function [6], and the risk of suicide is higher for those with a history of repeated episodes [7]. Self-harm bears a significant cost both to the individual and the healthcare system. Repetition of self-harm accrues substantial treatment costs with each hospitalisation; meanwhile, costs rise significantly in the 6 months following hospital attendance following the fifth and subsequent episodes, compared to the first [8].

A Cochrane review showed little evidence for the benefit of existing therapies for people who self-harm multiple times [9]. Therapies that have been studied are intensive, of long duration, require specialist therapists, and there is no published evidence of cost-effectiveness. The latest Cochrane review, NICE guidelines in the longer-term management of self-harm in adults (CG133) [10] and expert commentaries [11] all point to the need for new research to test the effectiveness of interventions in this population.

Despite the importance of reducing repetition, given that self-harm often occurs in challenging life circumstances and is associated with problems with mood, therapeutic approaches for individuals who repeatedly self-harm may be more effective if they focus on broader well-being, as opposed to a narrow focus on stopping self-harm. A therapeutic approach that works with service users to identify valued goals may be a more acceptable approach than therapies focused on reduction of the act itself ([12–14]; Copsey B, Bijsterveld P, Martin A, House A, Wright-Hughes A, Farrin A, et al.: Results of a feasibility study of the FReSH START intervention to improve quality of life and other outcomes in people who repeatedly self-harm (Function REplacement in repeated Self-Harm: Standardising Therapeutic Assessment and the Related Therapy), submitted). Up to now, the suggestion has been that repeated self-harm when associated with characteristics suggestive of borderline personality disorder [15], is best treated with specialist therapies like dialectical behavioural therapy or mentalisation-based therapy [16]. However, those with such characteristics are a relatively small proportion of the overall group of people who repeatedly self-harm and access to such therapies is often highly selective. Existing interventions for those who repeatedly seek hospital care following self-harm vary by region and service capacity but share some similarities. Many involve specialist practitioners and high treatment intensity and utilise a high volume of resources which contributes to long waiting times for patients. The scale of the problem in relation to resources in mental health services makes these an implausible first line solution. On-line group educational interventions, based on CBT or DBT principles, have been developed in some areas, in recognition of the large volume of people requiring help and the current limited resources. An intervention that improves the quality of life of people who repeatedly self-harm, that does not select on the basis of ‘personality disorder’ and that could be delivered without the need for expensive specialist services would potentially benefit tens of thousands of those who attend hospital each year. In addition to the personal and social benefits, a reduction in hospital attendance would alleviate pressure on secondary health services (local estimates are that each ED attendance and assessment costs £1200–1400), and a reduction in primary care attendance would reduce burden on general practitioners (GPs).

In earlier research, we modified three existing therapies, cognitive behavioural therapy (CBT), acceptance and commitment therapy (ACT), and psychodynamic interpersonal therapy (PIT), specifically for use with people who repeatedly self-harm (Copsey B, Bijsterveld P, Martin A, House A, Wright-Hughes A, Farrin A, et al.: Results of a feasibility study of the FReSH START intervention to improve quality of life and other outcomes in people who repeatedly self-harm (Function REplacement in repeated Self-Harm: Standardising Therapeutic Assessment and the Related Therapy), submitted). The modifications were based upon research we conducted to better understand the functions of self-harm [17], plus systematic reviews we undertook to better understand what factors help people to stop self-harming [18] and what people who self-harm value most about professionals/therapists who offer help [19]. All three therapies were adapted to include a focus on the underlying function or purpose of self-harm which was framed as a protective mechanism to cope with distressing thoughts and experiences (e.g. switching off distressing feelings, controlling suicidal thoughts, generating soothing feelings or conveying distress to others). The common factors of therapy [20, 21] were also given particular prominence in each of the three approaches, as although central to all therapeutic approaches, they are often overlooked in favour of particular therapeutic techniques. All three also shared a focus on helping an individual undertake activities they find important, to improve interpersonal interactions where relevant and to orient the individual’s choices around their own overarching goals and values. The trial was designed so that all three therapies were included in the same intervention arm of the trial. This was done for several reasons. First, although all three bona fide therapies retained their own specific factors, there was considerable overlap in their adapted approach for self-harm. Second, we considered that the potential differences in clinical meaningful effects between the three therapies would be of limited therapeutic value [22, 23]. Third, the majority of comparative psychotherapy studies are heavily underpowered and do not have sufficient power for detecting clinically relevant effect sizes [24]. A trial powered to detect any clinically meaningful differences between the three therapies would be prohibitively costly. Finally, as all three therapies are already practiced in NHS organisations, should the trial have a positive outcome, this would facilitate wider implementation than a focus on one therapy alone.

This paper describes the protocol for a randomised controlled trial (RCT) and forms part of a National Institute for Health Research (NIHR)-funded research programme, which seeks to develop and evaluate an intervention to improve outcomes in people who repeatedly self-harm (Copsey B, Bijsterveld P, Martin A, House A, Wright-Hughes A, Farrin A, et al.: Results of a feasibility study of the FReSH START intervention to improve quality of life and other outcomes in people who repeatedly self-harm (Function REplacement in repeated Self-Harm: Standardising Therapeutic Assessment and the Related Therapy), submitted).

### Objectives {7}

1. To evaluate the clinical and cost-effectiveness of a standard NICE-compliant care plus psychological intervention (modified PIT, CBT, ACT) versus standard NICE compliant care alone in people who present to emergency services with repeated self-harm
2. To investigate the processes of delivery, including acceptability and mechanisms of change, for the intervention

The primary statistical objective is to establish the effect of experimental therapy compared to usual care, on quality of life/psychological global distress (Clinical Outcomes in Routine Evaluation—Outcome Measure (CORE-OM)) at 12 months. Secondary objectives will investigate the effects of the intervention on secondary outcomes including repetition of self-harm hospital attendance, hopelessness, depression and social connectedness at 6 and 12 months. Economic evaluation outcomes include healthcare resource use costs and incremental cost per quality-adjusted life year (QALY) at 12 months and 5 years.

### Trial design {8}

The FReSH START trial is a prospective, pragmatic, multi-centre, superiority, individually (1:1) randomised controlled trial of standard care plus referral to intervention (one of three adapted therapies) vs standard care alone. It has an embedded internal pilot, economic and process evaluation.

## Methods: participants, interventions and outcomes

### Study setting {9}

Participants will be recruited from sixteen NHS mental health Trusts in the UK with some recruiting from multiple sites in their trusts (Leeds and York Partnership Foundation NHS Trust, Sheffield Health and Social Care NHS Foundation Trust, Greater Manchester Mental Health NHS Foundation Trust, Pennine Care NHS Foundation Trust (Oldham, Bury, Tameside), Tees, Esk and Wear Valleys NHS Foundation Trust, Kent and Medway NHS and Social Care Partnership Trust (Dartford, Medway), Lancashire and South Cumbria NHS Foundation Trust (Blackburn, Blackpool), Cambridgeshire and Peterborough NHS Foundation Trust, Gloucester Health and Care NHS Trust, Betsi Cadwaladr University Health Board, Northamptonshire Healthcare NHS Foundation Trust, Central and North West London NHS Foundation Trust, South West London & ST George’s Mental Health Trust, Avon and Wiltshire Mental Health Partnership NHS Trust, Southern Health NHS Foundation Trust (Southampton, Portsmouth), North Staffordshire Combined Healthcare NHS Trust). To ensure that our intervention is compatible with NHS practice, we will recruit through mechanisms which mirror NHS pathways. Thus, we will recruit participants who present at hospital ED as a consequence of self-harm or to services set up to divert patients from ED and who are seen by mental health services which operate in emergency settings (most often liaison mental health services).

### Eligibility criteria {10}

Participants who have attended the ED following an episode of self-harm and who have received a detailed psychosocial risk assessment from a mental health practitioner will be screened for the study.

Inclusion criteria:

- Aged 18 years or over;
- Registered with a GP in the catchment area of the mental health trust for the duration of the therapy;
- Presenting at ED as a consequence of self-harm, defined as intentional acts that directly harm a person’s own body. This includes methods like cutting, burning, scratching, banging or hitting parts of the body or interfering with wound healing, and it also includes self-poisoning, such as taking overdoses of drugs;
- Self-harm episode in the preceding three months that is at least their third episode in the preceding 12 months and their lifetime fourth or more episodes;
- Has mental capacity to provide fully informed written consent.

Exclusion criteria:

- Receiving, or having been referred to (and likely to receive this within the next 6 months), a specific psychological intervention that is similar to the trial intervention, or where a specific intervention is indicated for a related condition (e.g. anorexia nervosa) and would conflict with trial participation;
- Taken part in the FReSH START Feasibility study;
- Assessed by a mental health clinician as currently unsuitable for therapy (e.g. in crisis; actively suicidal, unable to tolerate therapy, i.e. past talking treatments have resulted in severe deterioration of mental state, has a diagnosis of schizophrenia, autism, or other form of severe mental illness that would be a contraindication for the talking treatments in this study);
- Lacking capacity to comply with study requirements;
- Insufficient proficiency in English to contribute to the data collection;
- Known risk of violence (for example reported by ED or liaison psychiatry staff);
- Researcher unable to contact potential participant within six weeks following self-harm event.

### Who will take informed consent {26a}

Potential participants who have attended ED for self-harm and have received a psychosocial assessment from a mental health practitioner will be introduced to the trial and screened for eligibility by the mental health practitioner. Eligible participants will be given summary information and a copy of the participant information sheet and asked for their permission for researcher contact. An additional suitability check will be carried out by a senior clinician who will review the potential participant’s health records and use their clinical expertise to assess any potential extraneous factors which may make trial participation unsuitable for the patient. The review will focus on the presence of patterns of behaviour that may impact on the ability of someone to participate in the trial, for example, patients with a history of violence towards clinical staff, a history of psychosis, who are already in therapy or have a diagnosis of autism. It will also check whether the potential participant has been referred to or offered psychological treatment, which is likely to start within the next 6 months.

Potential participants who provide consent to contact will be contacted by a researcher to explain the study in more detail and conduct a more detailed eligibility assessment. If the potential participant meets the eligibility criteria, the researcher will invite the potential participant to provide informed, written consent. The right of the participant to refuse consent without giving a reason will be respected. Electronic methods for documenting consent may be used.

Prior to consenting participants, their right to withdraw will be explained. They will be able to withdraw from different aspects of the study such as intervention, completion of questionnaires, receipt of text messages and/or access to medical records. They can withdraw for any reason at any time without their care being affected. Identifiable data already collected with consent will be retained and used in the final study analysis.

Following consent, participants will be registered onto the trial by the local researcher via a web-based system. Participants will then be able to complete baseline questionnaires (available in Additional file no. 1). Participants will then be randomised to standard care plus intervention or standard care alone.

### Additional consent provisions for collection and use of participant data and biological specimens {26b}

Participants will also be asked to consent to their anonymised data to be shared for future research projects. No biological samples will be collected.

## Interventions

### Explanation for choice of comparators {6b}

The comparator for the intervention is NICE compliant (CG133) standard care, available to both intervention and standard care only participants.

For the purposes of this trial, we base our definition of standard care upon NICE guidelines (CG 133), which stipulates the minimum intervention that the person who self-harms should receive a comprehensive assessment of needs and of risks. Information should be provided about possible strategies to help reduce self-harm, and consideration should be given to offering a psychological intervention. All service users who are eligible for the study will have been offered an integrated and comprehensive psychosocial assessment from a mental health practitioner of their needs and risks, with appropriate signposting or referral to relevant services, at the time of the person’s presentation to ED. Variation across sites is anticipated in the availability of and nature of signposted services. To ensure all study participants receive this standard care, potential participants will only be considered eligible for the study if they have already received a psychosocial assessment from a mental health practitioner, and the assessment will therefore occur prior to enrolment in the study.

To ensure all participants receive this intervention, we will only recruit people who have received a full psychosocial intervention from a mental health practitioner based in the emergency setting. In nearly all cases, this assessment will be conducted by a member of a mental health team.

### Intervention description {11a}

In addition to standard care, participants in the intervention arm will receive individual therapy in one of three therapeutic modalities: PIT, CBT and ACT. Therapies are modified to an assessment of self-harm functions component assessments: determining the functions of self-harm, identifying initial valued goals, choosing new strategies to reach valued goals. Participants will be offered up to twelve weekly sessions to be delivered over a maximum of 6 months, with the opportunity for 1–2 ‘top-up’ telephone contacts in months 6/7. Intervention therapy is to be considered complete after either twelve sessions have been delivered or if a mutually agreed ending to the sessions is reached. Each centre will offer two of the intervention trial therapies. Participants randomised to the intervention will be allocated within each centre to an intervention-therapist.

Therapy will be delivered by mental health professionals (mental health nurses, psychologists, occupational therapists, psychiatrists, counsellors, junior doctors) with significant experience in working with people who self-harm and managing risk, and who have received training in one of the modified therapies. Training will be delivered by therapy experts who have been involved with the development of the FReSH START components. Training will take place online over 3 days (3.5 for ACT) or equivalent, in groups of 3–12 trainees. All training will include role play with feedback. It is anticipated that at least two therapists per therapy per site will be trained.

The intervention will be delivered in accordance with the manual developed for each of the modified therapies and will be undertaken at appropriate Trust premises. Sessions may be delivered face to face, or remotely via telephone or video call, and will last between 45 to 50 min. Mode of delivery will be determined by current Trust practice, where possible taking into account therapist and participant preference. All sessions will be audio-recorded for use in supervision and fidelity assessment. Participants failing to attend or cancelling a session at the last minute will be able to rearrange appointments on a maximum of four occasions, after which no further appointments will be offered at the discretion of the therapist.

#### Modified therapeutic components

The three approaches overlap in several ways. They can be adapted to accommodate a range of functions for self-harm, going beyond emotion regulation functions alone, to accommodate functions that may be thought of as positive to the individual. They can also be adapted to place an individual’s overarching goals for their life at the heart of therapy.

The therapeutic assessment will focus upon elements of practice that people who self-harm find particularly helpful, including recognising the positive benefits that they experience from self-harm and the role it plays in their lives [17, 25]. The session will include a focus on exploring the personal goals and values of the participant, their interpersonal problems and difficulties, emotional distress and potential risk issues. Prominence will be given to formulation and understanding of the protective role that self-harm plays in their life, by undertaking a functional analysis of self-harm behaviour.

Subsequent sessions will focus on helping participants identify their own overarching goals and values and to consider how to orient their choices more closely around these, noticing patterns of thoughts, feelings, relationships and situations that are associated key choices or behaviours, including self-harm. Treatment may involve finding different ways to engage with strong or unpleasant feelings and impulses that could otherwise quickly lead to unhelpful choices or possibly self-harm.

### Cognitive behavioural therapy (CBT)

CBT draws on cognitive and behavioural approaches to understanding emotional distress. Cognitive approaches see the way we think about events as influencing our emotional reaction to them. Fundamental beliefs (a schema), once activated, give rise to negative thoughts which maintain emotional difficulties through a series of feedback loops, including behaviours. Behavioural approaches involve trying to understand the pattern of relationships between behaviours and emotional responses in terms of the function of the behaviour and then seeking to introduce new patterns of behaviour. These two theoretical approaches inform CBT treatment techniques, though different types of CBT may place more or less emphasis on one theoretical approach.

### Acceptance and commitment therapy (ACT) [26]

ACT is a newer form of cognitive behaviour therapy that aims to engender a quality called ‘psychological flexibility’, which can be defined as: ‘… the capacity to persist or to change behaviour in a way that includes conscious and open contact with thoughts and feelings (openness), appreciates what the situation affords (awareness), and serves one’s goals and values (engagement).’ Consequently, within an equal, warm relationship, a clinician will carefully offer a range of therapy methods and techniques to enhance psychological flexibility. For example, a clinician might help a person connect with their own over-arching goals and values by having conversations that enable a deeper understanding of how they want to be in their key relationships. A therapist may then help them find ways to include more activities that are consistent with these values into their everyday life. Willingness, self-compassion or de-centring exercises may then be explored as ways to approach the challenging thoughts and feelings that often accompany making important changes or doing new things.

### Psychodynamic interpersonal therapy (PIT)

This is a psychodynamic form of therapy which aims to manage feelings in the context of interpersonal relationships. It focuses upon interpersonal problems or ways of relating which may underpin symptomatic or problem scenarios. There is a strong focus on developing a strong therapeutic alliance from which interpersonal problems can be identified and solved. The different components of the model are as follows: (1) focus on feelings, (2) encourage the client to stay with feelings, (3) explore what associated thoughts, images, memories come to mind, (4) explore links or patterns in interpersonal relating that are problematic, (5) acknowledge these problematic patterns, (6) test out new ways of behaving both in the session with the client and in personal relationships outside. A goodbye letter is given to the client at the end of the therapy to summarise the work.

### Criteria for discontinuing or modifying allocated interventions {11b}

The intervention will be discontinued at the discretion of the therapist and clinical team if they judge the participant to be at increased risk of harm by continuing with therapy or if the participant wishes to withdraw. Participants can discontinue and/or withdraw from the study intervention at any time and will follow the same data collection follow-up schedule unless they withdraw from such aspects of the study.

The site clinician or therapist/supervisor may recommend that the participant be withdrawn from further active follow-up such as sending of SMS messages and/or questionnaires; however, it is expected for such instances to be rare and the default position will be for follow-up and data collection to continue.

### Strategies to improve adherence to interventions {11c}

To maximise intervention delivery, therapy can be delivered face to face or online. Therapist training, fidelity assessment and supervision aim to improve adherence to the intervention manual. All therapists will undertake a competency check as part of their training and as a prerequisite for delivering therapy.

Fidelity to each of the interventions will be measured, including fidelity to the self-harm adapted approach, and to each of the three psychological therapies (CBT, PIT and ACT). Inclusion of the key ingredients of the self-harm adaptation will be recorded for the first assessment session of each therapy.

Participants’ adherence to the intervention will be recorded by the number of sessions offered and attended, participants completing therapy and reasons for ending therapy. All data will be recorded by therapists via CRF.

All therapy sessions in the intervention arm of the trial will be audio-recorded. Fidelity assessments will be made via a therapist-reported checklist of content for all sessions and through researcher rated assessment of audio recordings of therapy sessions. These checklists are available as additional files (file numbers 2, 3 and 4). Fidelity will assess the following components of the intervention:

- Adaptations for working with people who self-harm in the initial assessment and subsequent sessions, specific to the FReSH START approach and common across the modified therapies
- Fidelity to the individual psychological therapeutic approach. In ACT, for example, therapy will be reviewed by an expert who is external to the trial using the Acceptance and Commitment Therapy Fidelity Measure (ACT-FM) [27]

The ratings of the FReSH START approach (available as Additional file no. 5) will be conducted on the initial sessions (sessions 1–3). A random sample of 10% of the first session of therapy, stratified by therapist and centre, will be reviewed and scored using the fidelity measure. The rater will have the option of listening to sessions two and three of the same therapy to complete their assessment as not all parts of the FReSH START approach may be completed in session one (for example safety planning may extend over sessions 1–3). We estimate 32–96 sessions will be rated representing 32 different therapists.

Assessment of the individual psychological therapeutic approach will be made using items from therapy-specific scales: the Sheffield Psychotherapy Rating Scale for PIT (Additional file no.2) and CBT (Additional file no. 3) and the ACT Fidelity measure for ACT (Additional file no.4). To assess competency of delivery for the first five therapists in all three therapy modalities, the fourth therapy session delivered will be appraised for its fidelity to the relevant therapy. These reviews will be conducted by the relevant member of the research team. If at this point the fidelity is deemed appropriate, no further assessment will take place. If, however, fidelity to the relevant therapy is not fulfilled, another check will take place after the seventh delivered session and if problems persist again after the ninth. If the fidelity check still fails after the ninth session, the therapist will not be allocated further trial participants, though consideration will be given regarding participants who may provide a particular challenge, making it difficult for the therapist to demonstrate adherence. If the competency assessment on the first five therapists of each modality all pass, the trial researchers can be assured that the training and assessment strategy for each modality is working. If persistent problems are identified, further therapists beyond the first five may have to be considered for competency.

Assessment of the individual psychological therapeutic approach will be made using items from therapy-specific scales: the Sheffield Psychotherapy Rating Scale for PIT and CBT and the ACT Fidelity measure for ACT. Assessment will be performed by a therapist with expertise in the relevant therapy. Approximately 5% of sessions will be randomly sampled, excluding the first three sessions as these are less likely to contain therapy specific interventions. An estimated 140 sessions will be rated.

Additionally, for the first five therapists in all three therapy modalities, the fourth therapy session delivered will be appraised for its fidelity to the relevant therapy. If at this point the fidelity is deemed appropriate, no further assessment will take place. If, however, fidelity to the relevant therapy is not fulfilled, another check will take place after the seventh delivered session and if problems persist again after the ninth. If the fidelity check still fails after the ninth session, the therapist will not be allocated further trial participants, though consideration will be given regarding participants who may provide a particular challenge, making it difficult for the therapist to demonstrate adherence. If the fidelity checks on the first five therapists of each modality all pass, the trial researchers can be assured that the training and assessment strategy for each modality is working. If persistent problems are identified, further therapists beyond the first five may have to be considered for assessment.

### Relevant concomitant care and intervention that are permitted or prohibited during the trial {11d}

Participants in both arms of the trial will be offered standard care as delivered in their study site. Participants must not receive another form of individual talking therapy during the intervention study period but can be referred to any other relevant services (e.g. community support groups, alcohol or drug services).

### Provisions for post-trial care {30}

Post-trial care of participants will be usual care offered by their healthcare organisation.

### Outcomes {12}

The primary outcome measure is the difference between the two randomised groups in mean CORE-OM score at 12 months [28]. The CORE-OM is a 34-item measure, widely used in primary and secondary care services in the NHS to assess levels of psychological global distress across four dimensions of well-being, symptoms (depression, anxiety, physical and trauma), functioning and risk [28]. Quality of life (QoL) is the most important patient-centred outcome as it can capture positive life changes—although reduction in the episodes of self-harm would be expected, one does not necessarily mediate the other. Secondary outcome measures include the Social Connectedness Scale (SCS) [29], the Beck Hopelessness Scale [30] and the PHQ-9 [31]. The SCS has been included as we argue that a valued goal is interpersonal in nature; therefore, achieving this through means other than self-harm will lead to more positive social connections which we know is associated with quality of life [32]. The PHQ-9 provides a reliable and valid measure of depression severity, and the Beck Hopelessness Scale as hopelessness has been shown to be a risk factor in suicidal behaviour [33].

- Secondary outcomes measures assessed at 6 and 12 months post randomisation (unless otherwise indicated) include difference in mean score on the CORE-OM at 6-months;
- Difference in the proportion of participants with reliable and clinically significant improvement on the CORE-OM (defined as a change of five or more in the clinical score and a change in score from the clinical to non-clinical range);
- Difference in mean score on the Beck Hopelessness Scale (BHS) [30], a 20-item self-report inventory used to measure feelings about the future, loss of motivation, and expectations;
- Difference in mean score on the Patient Health Questionnaire- 9 (PHQ-9) [31], a 9-item self-report measure of depression severity used to assess mental and emotional conditions;
- Difference in mean score on the Social Connectedness Scale—revised (SCS-R) [29], a 20-item scale used to measure social connectedness as a sense of belonging;
- Difference in rates of self-reported self-harm repetition measured monthly using questionnaires and also using monthly SMS messages for the 6-month follow-up period;
- Difference in the time to repetition of self-harm leading to hospital attendance using routinely collected data from Hospital Episode Statistics (A&E, emergency care, admissions, and outpatient datasets).

Health economic evaluation outcome measures.

- Quality-adjusted life years (QALYs) [34], generated via the CORE-6D preference-based measure based on six CORE-OM questions;
- Self-reported resource use from the NHS (primary and community care, medications) and patient (employment and financial costs due to self-harm) perspectives.

### Participant timeline {13}

This protocol follows the Standard Protocol Items: Recommendations for Interventional Trials (SPIRIT) guidelines and meets the SPIRIT checklist.

### Sample size {14}

The recruitment target is 565 participants equally distributed across standard care and intervention plus standard care. This will provide 90% power at 5% significance to detect a between-group difference of three points on CORE-OM (standardised effect size of 0.375) allowing for 25% loss to follow-up and design effect of 1.56. The design effect appropriately inflates the required sample size for an individually randomised controlled trial to account for clustering resulting from therapist-delivered intervention. The design effect assumes (i) cluster size of 11—on average each of 21 intervention therapists treat 15 patients, 11 provide follow-up data; (ii) coefficient of variation of 1.34 to account for variable numbers of patients per therapist (range 3–23) and (iii) intervention intra-cluster correlation coefficient (ICC) of 0.05 [35].

A similar design effect is assumed within the control arm: a wider range of therapists provide treatment as usual, resulting in fewer trial patients per standard care therapist (smaller cluster size) but potentially a higher ICC.

### Recruitment {15}

The trial plans to recruit from 12 to 14 sites over 36 months. The sample size of 565 requires that each site will recruit an average of 41 participants. Assuming the majority of sites recruit for a 24-month period due to staggered set-up, sites will recruit on average 2 participants per month.

An internal pilot will assess progression criteria on recruitment rates at nine months from the start of recruitment. Rates will be calculated from recruitment in each of the six sites in months 4 to 9 to allow time for recruitment to stabilise. The average number of participants recruited per month will be assessed as: red if < 12, amber if 12–19 and green if ≥ 20. Assuming six sites are open to recruitment, the criteria would be approximately equivalent to assessing monthly recruitment per site as: red if < 2, amber if 2–3.2, green if ≥ 3.3 participants per site per month. The internal pilot will inform a decision on continuation or modification of the trial, with an additional 9 months of recruitment anticipated thereafter. If the recruitment criterion is amber, we will review trial processes comprehensively, with the TSC and the funder, to see if modifications could be made to improve recruitment rates. If the recruitment criterion is red, the TSC and the funder will consider not continuing the trial.

## Assignment of interventions: allocation

### Sequence generation {16a}

Participants will be randomised to standard care plus intervention or standard care alone on a 1:1 basis. Randomisation will use minimisation with a random element, balanced on stratification factors of site, gender and baseline CORE-OM score (< 25 or ≥ 25, using derived score with a range of − 0 to 40).

### Concealment mechanism {16b}

The Clinical Trials Research Unit’s (CTRU) online 24-h computer-generated randomisation system will be used to generate the treatment allocation and ensure allocation concealment. Allocation information will only be released after the patient has been consented and recruited into the trial, and after all baseline measurements have been completed.

### Implementation {16c}

Randomisation will be requested by the study researcher at site using the CTRUs online randomisation system. An automated email will be sent from the randomisation service to the researcher responsible for recruitment and the site PI confirming randomisation and the participant’s allocation. Following randomisation, the researcher will contact participants and therapists to inform them of their allocation. A letter will also be sent to the participant’s GP informing them of their participation in the trial.

Participants allocated to standard care plus intervention will be further randomised to a trained therapist to receive one of the possible psychological therapies available for delivery within the site: CBT, PIT or ACT. Randomisation to therapist will be via simple randomisation stratified by site and will be performed concurrently within the CTRU online randomisation system. To allow for cases where the randomised therapist cannot deliver the intervention (e.g. due to participant preference on therapist gender, or therapist capacity), a backup randomised ordered list of all remaining therapists at site will also be generated and the researcher will liaise with the service lead and therapists in order until an allocated therapist is confirmed.

## Assignment of interventions: blinding

### Who will be blinded {17a} and procedure for unblinding {17b}

Due to the nature of the intervention, blinding of therapists and participants will not be possible therefore there is no requirement for emergency unblinding procedures in this study. Principal investigators and supervisors at sites will not be blinded since they may advise on risk incidents during therapy. Researchers involved in the organisation of therapy sessions will not be blinded. Follow-up questionnaires will be primarily conducted online or sent via post. However, some participants may prefer to complete their questionnaire by telephone. Where possible, staff members involved in collecting outcome data during follow-up will be blinded to allocation.

## Data collection and management

### Plans for assessment and collection of outcomes {18a}

Plans for the collection of baseline and outcome data are detailed in Table 1. Data collection forms can be found in Additional files 1 (Baseline Questionnaire) and 7, 8, 9 and 10 (3 m, 6 m, 9 m and 12 m Outcome Questionnaires).

**Table 1:**
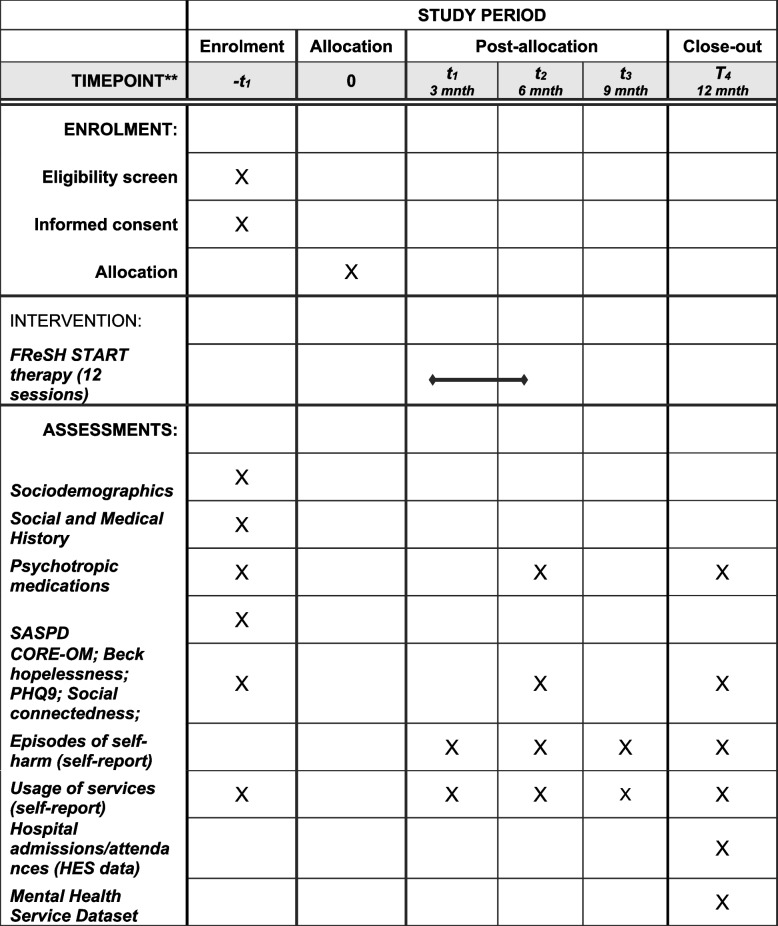
Participant timeline

The following questionnaires will be completed at baseline, following registration but prior to randomisation, by participant self-completion online (or researcher administration if preferred by participant):

- Clinical Outcomes in Routine Evaluation—Outcome Measure (CORE-OM) is a 34-item measure, used to assess levels of psychological global distress across four dimensions of well-being, symptoms (depression, anxiety, physical and trauma), functioning and risk [36]. The CORE-OM is acceptable to participants to complete and is widely used in primary and secondary care services in the NHS
- Hopelessness—Beck Hopelessness Scale is a 20-item self-report inventory and is used to measure feelings about the future, loss of motivation, and expectations [30, 37]
- Depression—PHQ-9 is a is a 9-item self-report measure of depression severity used to assess mental and emotional conditions, providing a reliable and valid measure of depression severity [38]
- Social connectedness—The Social Connectedness Scale-Revised is a 20-item scale used to measure social connectedness as a sense of belonging [29, 39]
- Self-reported resource use—primary and community care and medications and private financial burden due to self-harm (trial-specific).

Follow-up questionnaires will be completed at 6 and 12 months post-randomisation (to coincide with the anticipated end of therapy and at least 6 months after the end of therapy). The default method will be web-based completion, but paper copies can be posted out to participants if preferred. The following data will be collected at 6 and 12 months: Clinical Outcomes in Routine Evaluation—Outcome Measure (CORE-OM), Beck Hopelessness Scale, PHQ-9, the Social Connectedness Scale-Revised, self-reported episodes of self-harm, self-reported resource use—primary and community care and medications and private financial burden due to self-harm (trial-specific). In addition, participants will be asked to report on health resource use at 3 and 9 months, as recall over periods longer than 3 months may be suboptimal.

### Plans to promote participant retention and complete follow-up{18b}

To maximise participant retention and follow-up, the following measures will be implemented:

- Collection of data enabling multiple methods of data collation and contacting participants (email, post, phone);
- Those who do not complete the questionnaires will receive one automated reminder to prompt completion, followed by further reminders via telephone from a researcher who will also offer to support data collection over the telephone;
- A financial incentive will be offered for completion of 6-month questionnaires (£20 online voucher) and 12-month questionnaires (£40 voucher);
- A letter from the Lived Experience Group will also be sent to participants at 6- and 12-month time points with thanks and encouragement to continue engagement with the project;
- Researchers embedded within each healthcare organisation will deliver the trial and liaise with participants and therapists to facilitate retention.

### Data management {19}

Data will be recorded by researchers and site staff using a combination of trial-specific paper case report forms (CRFs) and remote data entry. Data recorded on paper CRFs, for example during face-to-face visits at baseline, or by therapists after therapy sessions, will be entered into a Remote Data Capture database by researchers or therapists at site or returned by post to Leeds CTRU, as per instructions supplied to sites.

Participant could opt to compete questionnaires online using REDCAP survey software, by post, or by telephone with a researcher. Participants who did not respond to the initial questionnaire were sent a reminder after 10 days. Participants who did not respond to the reminder questionnaire were contacted by telephone and were encouraged to complete the questionnaire with the option to complete it over the phone.

Relevant standard operating procedures, guidelines and work instructions in relation to data management, processing and analysis of data will be followed. At the end of the study, data will be securely archived at the University of Leeds for a minimum of 5 years. Data held by the University of Leeds will be archived in the Leeds Sponsor archive facility, and site data and documents will be archived at site. Following authorisation from the sponsor, arrangements for confidential destruction will then be made.

Data collected through observations (field notes and observational records, audio recorded interviews, summaries of documentary analysis) and reflective reports will be anonymised and stored at the Leeds Institute of Health Sciences, University of Leeds.

### Confidentiality {27}

All information collected during the course of the study will be kept strictly confidential. Information will be held securely on paper and electronically at the CTRU. The CTRU and LIHS will comply with all aspects of the 2018 Data Protection Act. Participant name, address and telephone number will be collected when a participant is registered into the trial, but all other data collection forms that are transferred will be coded with a trial number and will include two participant identifiers, usually the participant’s initials and date of birth. All records that contain personal identifiers, such as informed consent and contact details forms, will be stored separately from study records identified by Trial ID code.

Appropriate processes will be put in place for the transfer, storage, restricted access and disposal of personal information. To ensure confidentiality of the data collected when published, fictitious site names and pseudonyms or study numbers not linked to sites or persons will be used. All identifiable data such as research site names, address, date of birth and participants’ names will be removed.

### Plans for collection, laboratory evaluation and storage of biological specimens for genetic or molecular analysis in this trial/future use {33}

This is not applicable; no specimens were collected.

## Statistical methods

### Statistical methods for primary and secondary outcomes {20a}

A detailed statistical analysis plan will be finalised and agreed by the research team prior to analysis.

A single final analysis is planned after the trial is closed to recruitment and follow-up and when the full database has been cleaned and locked. Analyses will be completed by the CTRU statisticians using SAS version 9.4.

The primary analysis of CORE-OM will use mixed multilevel linear regression with repeated measures (6- and 12-month outcome) adjusted for randomisation stratification factors and baseline value, with therapist treated as a random intercept. Estimated mean treatment differences at each time point will be reported with 95% confidence interval, *p*-values and ICCs. A two-sided 5% significance level will be used for statistical endpoint comparisons, unless otherwise specified.

Secondary outcomes, reliable and clinically significant improvement on the CORE-OM, hopelessness, depression and social connectedness will be analysed using the same approach, i.e. mixed multilevel linear or logistic regression, depending on the type of outcome variable.

Secondary outcomes repetition of self-harm leading to hospital and self-reported repetition of self-harm will be analysed separately.

Cox proportional hazards, accounting for randomisation stratification factors and random therapist effect (multilevel survival frailty model) [40], will be used to test for differences in the time to first repetition and the proportion of participants attending hospital for self-harm at 6 and 12 months. Hazard ratios and corresponding 95% CIs will be presented. If a participant is lost to follow-up, they will be treated as censored. Kaplan–Meier curves will be constructed for each group.

Recurrent event analysis incorporating the timing and cumulative number of hospital attendances for self-harm will be used to test for differences in the rate of recurrent events. Estimated treatment differences will be reported as the hazard ratio with 95% confidence interval and *p*-values.

Self-reported self-harm will be summarised at each follow-up time point (monthly to 12 months).

The number of episodes of self-reported self-harm at 6 and 12 months from questionnaires (supplemented by monthly SMS data) will be analysed using Poisson regression, if appropriate. This will be a secondary method for the assessment of self-harm repetition. The choice of analysis method for self-reported self-harm will be informed by the data from the feasibility study.

### Interim analyses {21b}

No formal interim analyses are planned. An internal pilot analysis will assess recruitment progression criteria 9 months from the start of recruitment; participants from this analysis will be included in the final trial analysis.

Safety data will be summarised for the DMEC during the trial; however, this will not include any primary or secondary outcome data. Follow-up rates and intervention uptake will be monitoring closely by the PSC and DMEC throughout the trial.

## Methods for additional analyses {20b}

### Subgroup analyses

For the primary outcome, exploratory analysis will compare mean scores across the three therapy modalities. When comparing individual therapies against usual care, a significance level of 1.7% will be used to adjust for multiple testing.

For the CORE-OM, a mixed model analysis will be used as in the primary outcome analysis with the addition of an interaction term between baseline SASPD and treatment and the inclusion of baseline SASPD as a covariate. For time to first repetition of self-harm leading to hospital attendance, Cox proportional hazards will be used with the addition of an interaction term between baseline SASPD and treatment and the inclusion of baseline SASPD as a covariate.

### Health economics analysis

The health economic evaluation will assess the cost-effectiveness of the self-harm-focused psychological therapy intervention relative to standard care following methods stated in the NICE methods and processes manual [41]. We will conduct both a within-trial and a decision analytic model-based cost-effectiveness analysis. The primary analysis will adopt an NHS and personal social services perspective. A sensitivity analysis will include societal costs related to patient out of pocket expenses and productivity losses. Health outcomes will be measured as quality-adjusted life years (QALYs). Intervention costs will be calculated based on the time required for therapists to deliver therapy, in addition to costs related to training and supervision.

For the within-trial analysis, healthcare costs will be obtained from hospital episode statistics data (NHS digital), combined with primary care, medication and community costs obtained from participant reported resource use questionnaires collected at 3, 6, 9, and 12 months. Health-related quality of life data will be obtained from participant responses to the CORE-6D, derived from 6 questions in the CORE-OM each with three severity responses. Health states from the CORE-6D will be mapped to UK time trade off utility values using an algorithm developed by Mavranezouli et al. [42]. QALYs will be calculated by combining health-related quality of life (HRQoL) with Office of National Statistics (ONS) mortality data. Missing data techniques consistent with the statistical modelling such as multiple imputation will be adopted to account for missing data.

The model-based analysis will use a cohort Markov model to extend results beyond the trial time horizon. Due to uncertainty in the long-term data, we will adopt a 5-year time horizon for the base case model and explore extensions (e.g. 10 years, 20 years and lifetime) in sensitivity analyses. The model will be parameterised using data collected during the trial and from published literature. All costs and benefits beyond 12 months will be discounted at 3.5% per annum.

Results will be reported in accordance with the Consolidated Health Economic Evaluation Reporting Standards (CHEERS) guidelines with cost-effectiveness summary portrayed as incremental net health benefit (INHB), incremental net monetary benefit (INMB) and incremental cost-effectiveness ratio (ICER) with maximum willingness to pay (MWtP) thresholds set over the usual NICE range £20,000–£30,000 per QALY. Uncertainty will be explored with univariate and probabilistic sensitivity analysis (PSA) with results reported on the cost-effectiveness plane (CEP) and as a cost-effectiveness acceptability curve (CEAC) over a MWtP range of £0 to £50,000 per QALY.

### Process evaluation analysis methods

A concurrent mixed methods process evaluation will be conducted to explore the implementation and acceptability of the intervention, to understand the mechanisms of change, and to explore the mediating role of context [43]. We will collect quantitative process data assessing the fidelity (as described above) and dose and acceptability of the intervention as well as trial outcome data and stakeholder characteristics. We will also conduct qualitative interviews to explore stakeholder experiences, the context, acceptability and implementation. Information about the organisation context and wider externalities will be collected from interviews, site visits and meetings with study site clinical and research teams.

We will conduct one to one interviews (telephone or face to face) with participants (*n* = 30) purposively sampled on age, gender, method of self-harm and therapy modality, to explore their experience of receiving the therapy and perceived impact on social and psychological well-being. All participants will be given the option to be invited to interview when they consent to the study. Specific participant information sheets and consent forms will be provided. Insights into mechanisms of action, intervention delivery and context will be made to help in the interpretation of trial findings and give implementation advice for any subsequent take up of intervention into MHLT provision. A purposive sample 20–30, depending upon variability, of therapists by therapy modality, study site and characteristics will be invited to be interviewed. Service managers will also be interviewed to explore how the intervention has impacted on the wider team and delivery of regular services.

Interviews will be audio recorded, using a digital audio recording device, and transcribed. During transcription, any potentially identifying information that may be contained in the interview discussions will be removed. Qualitative analysis will occur concurrently with data collection to allow for insights generated or new theories offered to be explored and tested in subsequent interviews. Analysis will focus on evidence to explore how the intervention is received, evidence to develop and refine theories of how the intervention might work and on evidence to suggest how context might affect how the intervention works in practice.

The quantitative component of the evaluation includes data from the fidelity checks described in the “Strategies to improve adherence to interventions {11c}” section. These will be combined to provide contextual data about study sites as well as providing descriptive characteristics of therapists to help understand the relationship between adherence to the intervention and the acceptability to participants that will be explored in qualitative interviews.

Several substantial amendments have been made to the trial protocol, including the introduction of incentive vouchers for completion of questionnaires at 6 and 12 months, a letter from the Lived Experience Group (LEG) to encourage follow-up completion, defining cut-off points for therapy completion, therapist assessment of trial readiness, additional fidelity checks at sessions 4, 5, and 9 and addition of new documents including a recruitment poster and a letter to potential participants confirming the unsuitability of the trial for them. Site PIs, local researchers and participants (where relevant) will be informed of all amendments following ethical and regulatory approvals. Trial registries and journals will be informed as appropriate.

### Methods in analysis to handle protocol non-adherence and missing data {20c}

All analyses and data summaries will be conducted on the intention-to-treat (ITT) population which is defined as all participants randomised regardless of non-compliance with the protocol or withdrawal from the study.

If appropriate, a CACE analysis using the instrumental variable approach will be conducted to estimate the treatment effect for the primary outcome when accounting for non-compliance with the intervention.

Missing data is expected; the reasons (e.g. due to loss to follow-up, illness or at random) and mechanisms for missing data will be explored, and the proportion of missing data will be compared between the intervention and control groups and by participant baseline characteristics. The sample size calculation allowed for 25% loss to follow-up for the primary outcome.

For primary and secondary outcomes, a multiple imputation model will be built under the missing at random assumption with imputation at the individual participant level. Sensitivity analyses of the primary endpoint will be conducted to assess the impact of missing data, the choice of imputation model and the missing at random assumption, as appropriate.

### Plans to give access to the full protocol, participant-level data and statistical code {31c}

Data will be available upon request and subject to approval by the Leeds CTRU and FRESHSTART CI. A legally binding data sharing agreement will be required prior to access/sharing of any study data.

## Oversight and monitoring

### Composition of the coordinating centre and trial steering committee {5d}

The *Programme Steering Committee (PSC)* will co-provide overall supervision of the programme grant and trial, in particular, study progress, adherence to protocol, participant safety and consideration of new information. It will include an independent chair, no fewer than two other independent members with appropriate clinical and statistical expertise, and a patient representative. The CI and other members of the Programme Management Group may attend the PSC meetings and present and report progress. The committee will meet once during the set-up period and at least annually thereafter for the duration of the study.

The* Programme Management Group* (PMG) will oversee the whole FReSH START Programme Grant, comprising the chief investigator, programme manager, co-applicants, co-investigators, and NHS host.

The* Trial Management Group* (TMG) comprises the chief investigator, the programme manager, key co-applicants, research fellows and CTRU staff. The TMG will meet at key points during the study to oversee the study including the set-up, on-going management, promotion of the study and the results.

A trial monitoring plan will be developed and agreed by the TMG and PSC based on the trial risk assessment which will consider the safety or physical or mental integrity of the trial participants and the scientific value of the research.

The* Lived Experience Group* (LEG) provides advice and accountability from people with lived experience of self-harm. The group will meet at regular intervals over the trial period based on group preferences and whenever research activity necessitates their input. Members will advise on study processes and materials, guide our communication with stakeholders and wider audiences and contribute to process evaluation data analysis and the development of dissemination plans and materials.

The trial is managed on a day-to-day basis by the CIs, the research fellows and core team at the Leeds CTRU who will regularly meet to discuss the study. They will be responsible for the set-up of the study, including gaining ethical and R&D approval, appointment of additional researchers if required, management and overall supervision of the study team, collection and analysis of data and drafting publications. The chief investigator will be responsible for the day-to-day running of study.

The CTRU will be responsible for the following: registration, randomisation system, database development and provision, CRF design, data management and quantitative design, analysis and reporting.

### Composition of the data monitoring committee, its role and reporting structure {21a}

A Data Monitoring and Ethics Committee (DMEC) will be convened to monitor data collected during the study and make recommendations to the PSC on whether there are any ethical or safety reasons as to why the trial should not continue. It will consist of an independent chair, an independent statistician and an independent clinician. The DMEC will meet annually as a minimum.

### Adverse event reporting and harms {22}

A serious adverse event (SAE) is any untoward medical occurrence that results in death, is life-threatening, requires inpatient hospitalisation or prolongation of existing hospitalisation, results in persistent or significant disability/incapacity, consists of a congenital anomaly or birth defect.

The National Research Ethics Service (NRES) defines related and unexpected SAEs (RUSAEs) as follows:

- ‘Related’—that is, it resulted from administration of any research procedures; and
- ‘Unexpected’—that is, the type of event is not listed in the protocol as an expected occurrence.

All related/unexpected SAEs occurring from the date of consent up to 12 months post-randomisation will be recorded on the related/unexpected serious adverse event (RUSAE) form and faxed to the CI within 24 h of the clinical research staff becoming aware of the event. All related/unexpected SAEs will be reviewed by the chief investigator, notified to the sponsor within one working day, and are subject to expedited reporting to the main REC by the CTRU on behalf of the chief investigator within 15 days. Events will be followed up until the event has resolved or a final outcome has been reached.

The following events are expected within the study population and will be collected from date of consent until 12 months post-randomisation: hospital admissions and re-admissions, life-threatening repeated self-harm not leading to hospital admission, death (including suicide).

In this population, the expected rate of deaths for those who self-harm is approximately 60–100 times that of the population as a whole; thus, it is possible that some people may die as a consequence of self-harm during the course of the study. As deaths are more likely within this population, they will not be subject to expedited reporting to the main REC, unless the PSC and DMEC advises that the frequency of self-harm related and/or all deaths observed within the trial population is significantly higher than that expected in the general self-harm population. All deaths occurring from the date of consent up to 12 months post-randomisation will be recorded on the death form and reported. The Data Monitoring and Ethics Committee (DMEC), funder, and sponsor will be informed of deaths (with the data available at the time) within 1 month of reporting by site.

Hospital admission details will be obtained by regular extracts from centralised hospital records via Hospital Episode Statistics from NHS digital (supplemented by researcher review of local hospital records).

### Frequency and plans for auditing trial conduct {23}

Site monitoring and audit will be informed by a risk-based approach, with quality assurance audit undertaken by, or on behalf of, the sponsor if there is cause to do so.

### Plans for communicating important protocol amendments to relevant parties (e.g. trial participants, ethical committees) {25}

Protocol amendments will be subject to review by the research ethics committee and approved changes communicated to trial investigators, the PSC and study site research teams.

### Dissemination plans {31a}

Findings will be published via peer-reviewed journal articles, reporting to the funder, national and international conferences and public, patient, participant and NHS stakeholder groups via written and in person dissemination events. A publication policy and dissemination plan will be agreed by members of the PMG.

## Discussion

This paper describes a protocol for a proposed randomised controlled trial to investigate whether modified therapies can improve quality of life for people who repeatedly self-harm and to test the cost-effectiveness of this intervention. Currently, there are few tested interventions deliverable in the NHS to support this population.

Particular strengths of the trial are the use of mental health practitioners who would not usually have the opportunity to deliver therapy but whose experience of working with people who self-harm can provide the level of risk management required for safe therapy delivery. Increasing the skills of the existing NHS workforce may increase implementation into clinical practice. At the heart of this study is the experience of people with lived experience of self-harm. This novel intervention and associated research components were designed with significant input from people with lived experience. A diverse panel continue to be a core part of the study team advising on real time changes to the trial as well as participating in the analysis of process evaluation data. This will maximise the intervention uptake and potential effectiveness as well as increasing the impact of dissemination strategies.

The study team have encountered multiple recruitment and implementation challenges which we have been able to address in this protocol to minimise impact on recruitment. There is variation in reported eligible attendances at ED and we know that there may be variability of staffing numbers over any 24 h period which may affect the site’s capacity to recruit.

All clinical teams have reported an increase in staff turnover in the later stages and period post-pandemic. This has been anecdotally attributed to wider NHS workforce retention issues as well as mental and physical stress following the COVID-19 pandemic. These pressures have led to difficulties in sustaining the engagement of the clinical/recruiting team and in communicating the study to ensure staff confidence in introducing the study to potential participants. To address these, additional materials have been developed (patient facing posters, guidance notes for liaison team members, crib sheet for clinicians, reminder cards etc.) as well as additional training to support clinical teams to introduce the study and screen potential participants.

There are also potential challenges in the retention and confidence of trained staff to deliver the intervention, potentially exacerbated by administrative delays to site opening. The trial had planned to train approximately 21 therapists across 12 sites to deliver therapy; however, the retention of trained staff to deliver the intervention in the feasibility suggested this would remain a challenge in the RCT and some sites only having capacity for therapists to take one participant at any one time rather than two. During feasibility, therapists reported a decline in confidence if there was a long gap between training and commencing delivery. Therapists who were less confident reported adhering more closely to the manual and using it as a prompt during sessions. Multiple training sets are planned to train many more therapists than anticipated and to ensure opportunities for brief refresher sessions, and therefore therapist confidence, is maximised.

## Trial status

The trial opened to recruitment in October 2021 and planned to continue for 18 months until March 2023; however, due to multiple recruitment and implementation challenges, a recruitment extension has been granted and recruitment is anticipated to continue for a further 18 months.

As a result of an increased number of therapists trained and delivering the intervention than assumed within our sample size calculation, we anticipate a gain in power and/or a reduction in the number of participants required to maintain a suitably powered trial. We therefore planned to re-estimate the required sample size based on updated clustering assumptions once sufficient data are available on intervention delivery, when 300 participants have been recruited or (as requested by the funder) in October 2023 whichever is sooner. Clustering effects were re-estimated in Autumn 2023 as planned. In February 2024, the funder supported by the Programme Steering Committee agreed a revised recruitment target of a minimum of 348 participants over a total of 36 months. As of 30 June 2024, we had recruited 266 participants across 15 centres. The trial team are still in discussions with new potential centres.

Protocol number and date: 6.0 24 April 2023.

Recruitment started: October 2021.

Anticipated recruitment end: 30 September 2024.

### Supplementary Information

Additional file 1. Baseline Questionnaire.

Additional file 2. PIT Fidelity Checklist.

Additional file 3. CBT Fidelity Checklist.

Additional file 4. ACT Fidelity Checklist.

Additional file 5. FReSH START component fidelity checklist.

Additional file 6. 3 month Outcome Questionnaire.

Additional file 7. 6 month Outcome Questionnaire.

Additional file 8. 9 month Outcome Questionnaire.

Additional file 9. 12 month Outcome Questionnaire.

## Data Availability

Data supporting this work are available on reasonable request. Individual participant data (with any relevant supporting material, e.g. data dictionary, protocol, statistical analysis plan) for all trial participants (excluding any participant opt-outs) will be made available for secondary research purposes at the end of the trial, i.e. when all primary and secondary endpoints have been met and all key analyses are complete. Requests to access trial data should be made to CTRU-DataAccess@leeds.ac.uk in the first instance. Requests will be reviewed (based on the above principles) by relevant stakeholders, based on the principles of a controlled access approach. No data will be released before an appropriate agreement is in place setting out the conditions of release.

## Abbreviations

AE: Adverse event
ACT: Acceptance and commitment therapy
BHS: Beck Hopelessness Scale
CBT: Cognitive behavioural therapy
CEAC: Cost-effectiveness acceptability curve
CEP: Cost-effectiveness plane
CHEERS: Consolidated Health Economic Evaluation Reporting Standards
CLRN: Comprehensive Local Research Network
CORE-OM: Clinical Outcomes in Routine Evaluation – Outcome Measure
CRF: Case report form
CTRU: Clinical Trials Research Unit
CTS: Cognitive Therapy Scale
DBT: Dialectical behaviour therapy
ED: Emergency department
GCP: Good Clinical Practice
GDPR: General Data Protection Regulation
GP: General practitioner
HRQoL: Health-related quality of life
INHB: Incremental net health benefit
INMB: Incremental net monetary benefit
ICER: Incremental cost-effectiveness ratio
ITT: Intention-to-treat
LIHS: Leeds Institute of Health Sciences
LTHT: Leeds Teaching Hospitals Trust
LYPFT: Leeds and York Partnership NHS Foundation Trust
REC: Research ethics committee
MWtP: Maximum willingness to pay
NHS: National Health Service
NICE: National Institute for Health and Clinical Excellence
NIHR: National Institute for Health Research
NRES: National Research Ethics Service
PIT: Psychodynamic interpersonal therapy
PHQ9: Patient Health Questionnaire 9
PMG: Programme Management Group
PSA: Probabilistic sensitivity analysis
PSC: Programme Steering Committee
QALY: Quality-adjusted life year
REC: Research ethics committee
REDCap: Research Electronic Data Capture
RCSI: Reliable and clinically significant improvement
RCT: Randomised controlled trial
R&D: Research and development
RUSAE: Related, unexpected serious adverse event
SASPD: Standardised Assessment of Severity of Personality Disorder
SAE: Serious adverse event
SC: Standard care
SH: Self-harm
SMS: Short Message Service
SOP: Standard operating procedure
SSA: Site-specific assessment
TMG: Trial Management Group
