# Supplementary material for: Randomised controlled trial of a psychotherapeutic intervention to improve quality of life and other outcomes in people who repeatedly self-harm: FReSH START study protocol
Source: Trials. 2024 Aug 26;25:564. doi: 10.1186/s13063-024-08369-2 (PMC11346196; doi:10.1186/s13063-024-08369-2)
Supplement: Supplementary file 2 — Additional file 2. PIT Fidelity Checklist. [file 13063_2024_8369_MOESM2_ESM.pdf]

ISRCTN 73357210

FReSH START
Rethinking support for self-harm

# FORM 36



# Therapy Fidelity Assessment – PIT

Participant ID number *(you can obtain this from the audio file label)* Site Code | Trial No

Rater name

Date of session being rated Day Month Year

Session being rated 1 ☐ 2 ☐ 3 ☐ 4 ☐ 5 ☐ 6 ☐ 7 ☐ 8 ☐ 9 ☐ 10 ☐ 11 ☐ 12 ☐

## PIT-specific Adherence

1. STATEMENTS: Did the therapist use statements, rather than questions to explore feelings, bring feelings into the ‘here and now’ and ‘stay with feelings’?

| 1 | 2 | 3 | 4 | 5 | 6 | 7 | 8 |
|---|---|---|---|---|---|---|---|
| Not at all | | Some | | Quite a lot | | Considerably | Not applicable |

2. UNDERSTANDING HYPOTHESES: Did the therapist offer statements of empathic understanding in response to cues?

| 1 | 2 | 3 | 4 | 5 | 6 | 7 | 8 |
|---|---|---|---|---|---|---|---|
| Not at all | | Some | | Quite a lot | | Considerably | Not applicable |

3. NEGOTIATING STYLE: Did the therapist express his/her views concerning the patient’s experiences and circumstances as tentative statements, open to correction, and inviting elaboration and feedback?

| 1 | 2 | 3 | 4 | 5 | 6 | 7 | 8 |
|---|---|---|---|---|---|---|---|
| Not at all | | Some | | Quite a lot | | Considerably | Not applicable |

4. THERAPY RATIONALE: Did the therapist provide a rationale which emphasised that working on understanding and changing the client’s characteristic patterns of feeling and action in relationships would help overcome the client’s difficulties and symptoms?

| 1 | 2 | 3 | 4 | 5 | 6 | 7 | 8 |
|---|---|---|---|---|---|---|---|
| Not at all | | Some | | Quite a lot | | Considerably | Not applicable |

5. CUE BASIS: Did the therapist explicitly pick up or acknowledge cues (verbal and non-verbal) when supplied by the client?

| 1 | 2 | 3 | 4 | 5 | 6 | 7 | 8 |
|---|---|---|---|---|---|---|---|
| Not at all | | Some | | Quite a lot | | Considerably | Not applicable |

Completed by

Date Day Month Year

*Form continues on next page ►►*

*Prior to returning this form to CTRU you must make a copy of the form and any amendments for retention at site. CTRU, University of Leeds (please see Investigator Site File for full contact details).*

| For office use only | Computerised | | Verified/Checked | |
|---|---|---|---|---|
| | *Date* | *Initials* | *Date* | *Initials* |

# Therapy Fidelity Assessment – PIT

## PIT-specific Adherence *(Continued)*

6. METAPHOR: Did the therapist encourage and elaborate the client's use of metaphor or use metaphor themselves to deepen feeling or understanding?

| 1 | 2 | 3 | 4 | 5 | 6 | 7 | 8 |
|---|---|---|---|---|---|---|---|
| Not at all | | Some | | Quite a lot | | Considerably | Not applicable |

7. FOCUSSING: Did the therapist focus on the 'here and now' experience of the client in the session, encouraging the client to stay with feelings before any attempt to 'explain' them?

| 1 | 2 | 3 | 4 | 5 | 6 | 7 | 8 |
|---|---|---|---|---|---|---|---|
| Not at all | | Some | | Quite a lot | | Considerably | Not applicable |

8. EXPLORATION OF FEELINGS: Did the therapist help the client to explore her/his feelings related to an interpersonal relationship?

| 1 | 2 | 3 | 4 | 5 | 6 | 7 | 8 |
|---|---|---|---|---|---|---|---|
| Not at all | | Some | | Quite a lot | | Considerably | Not applicable |

Does the average rating score demonstrate fidelity to PIT? ☐ Yes ☐ No

Completed by ____________ Date Day | Month | Year

*Last Page* ■

*Prior to returning this form to CTRU you must make a copy of the form and any amendments for retention at site. CTRU, University of Leeds (please see Investigator Site File for full contact details).*

| For office use only | Computerised | | Verified/Checked | |
|---|---|---|---|---|
| | Date | Initials | Date | Initials |
