# Supplementary material for: Randomised controlled trial of a psychotherapeutic intervention to improve quality of life and other outcomes in people who repeatedly self-harm: FReSH START study protocol
Source: Trials. 2024 Aug 26;25:564. doi: 10.1186/s13063-024-08369-2 (PMC11346196; doi:10.1186/s13063-024-08369-2)
Supplement: Supplementary file 3 — Additional file 3. CBT Fidelity Checklist. [file 13063_2024_8369_MOESM3_ESM.pdf]

ISRCTN 73357210

FReSH START
Rethinking support for self-harm

# FORM 35



# Therapy Fidelity Assessment – CBT

Participant ID number (you can obtain this from the audio file label) — Site Code | Trial No

Rater name

Date of session being rated — Day Month Year

Session being rated: ☐ 1 ☐ 2 ☐ 3 ☐ 4 ☐ 5 ☐ 6 ☐ 7 ☐ 8 ☐ 9 ☐ 10 ☐ 11 ☐ 12

## CBT-specific Adherence

1. RELATIONSHIP OF THOUGHTS AND FEELINGS: Did the therapist encourage the client to relate affective states that the client had experienced (OR will experience in the future) to the client's ongoing thoughts?

| 1 | 2 | 3 | 4 | 5 | 6 | 7 | 8 |
|---|---|---|---|---|---|---|---|
| Not at all | | Some | | Quite a lot | | Considerably | Not applicable |

2. REPORTING COGNITIONS: Did the therapist ask the client to report specific thoughts (as verbatim as possible) that the client experienced either in the session OR in a situation which occurred prior to the session?

| 1 | 2 | 3 | 4 | 5 | 6 | 7 | 8 |
|---|---|---|---|---|---|---|---|
| Not at all | | Some requests | | Quite a lot | | Considerable number of requests | Not applicable |

3. EXPLORING PERSONAL MEANING: Did the therapist probe for beliefs related to a thought the client reported in order to explore the personal meaning associated with the client's initial thought?

| 1 | 2 | 3 | 4 | 5 | 6 | 7 | 8 |
|---|---|---|---|---|---|---|---|
| Not at all | | Some | | Quite a lot | | Considerably | Not applicable |

4. PLANNING / PRACTICING ALTERNATIVE BEHAVIORS: Did the therapist work with the client to plan OR to practice alternative overt behaviors for the client to utilize outside of therapy?

| 1 | 2 | 3 | 4 | 5 | 6 | 7 | 8 |
|---|---|---|---|---|---|---|---|
| Not at all | | Some | | Quite a lot | | Considerably | Not applicable |

5. HOMEWORK ASSIGNED: Did the therapist or client develop one or more specific assignments for the client to engage in between sessions?

| 1 | 2 | 3 | 4 | 5 | 6 | 7 | 8 |
|---|---|---|---|---|---|---|---|
| Not at all | | Some | | Quite a lot | | Considerably | Not applicable |

Completed by | Date — Day Month Year

Form continues on next page ►►

*Prior to returning this form to CTRU you must make a copy of the form and any amendments for retention at site. CTRU, University of Leeds (please see Investigator Site File for full contact details).*

| For office use only | Computerised | | Verified/Checked | |
|---|---|---|---|---|
| | Date | Initials | Date | Initials |

# Therapy Fidelity Assessment – CBT

## CBT-specific Adherence *(Continued)*

6. SCHEDULING / STRUCTURING ACTIVITIES: Did the therapist work with the client to schedule OR structure one or more specific activities for the purpose of increasing the likelihood that the client will initiate OR follow through on those activities?

| 1 | 2 | 3 | 4 | 5 | 6 | 7 | 8 |
|---|---|---|---|---|---|---|---|
| Not at all | | Some | | Quite a lot | | Considerably | Not applicable |

7. SELF-MONITORING: Did the therapist encourage the client to record feelings, activities, or events between sessions OR review the client's records of feelings, activities, or events?

| 1 | 2 | 3 | 4 | 5 | 6 | 7 | 8 |
|---|---|---|---|---|---|---|---|
| Not at all | | Some | | Quite a lot | | Considerably | Not applicable |

8. MANIPULATING BEHAVIOR VIA CUES OR CONSEQUENCES: Did the therapist help the client to arrange for cues (i.e. stimulus control) OR consequences (i.e. reinforcement or punishment) for the client's specific thoughts or behaviors in order to manipulate the occurrence of those behaviors?

| 1 | 2 | 3 | 4 | 5 | 6 | 7 | 8 |
|---|---|---|---|---|---|---|---|
| Not at all | | Some | | Quite a lot | | Considerably | Not applicable |

Does the average rating score demonstrate fidelity to CBT? ☐ Yes ☐ No

Completed by ______ Date (Day / Month / Year) ______ *Last Page* ■

*Prior to returning this form to CTRU you must make a copy of the form and any amendments for retention at site. CTRU, University of Leeds (please see Investigator Site File for full contact details).*

| For office use only | Computerised | | Verified/Checked | |
|---|---|---|---|---|
| | Date | Initials | Date | Initials |
