# Supplementary material for: Randomised controlled trial of a psychotherapeutic intervention to improve quality of life and other outcomes in people who repeatedly self-harm: FReSH START study protocol
Source: Trials. 2024 Aug 26;25:564. doi: 10.1186/s13063-024-08369-2 (PMC11346196; doi:10.1186/s13063-024-08369-2)
Supplement: Supplementary file 4 — Additional file 4. ACT Fidelity Checklist. [file 13063_2024_8369_MOESM4_ESM.pdf]

FReSH START
Rethinking support for self-harm

FORM 34

Therapy Fidelity Assessment – ACT

Participant ID number *(you can obtain this from the audio file label)* Site Code | | | | | Trial No | | | | |

Rater name

Date of session being rated Day | | Month | | Year | | | |

Session being rated

1	2	3	4	5	6	7	8	9	10	11	12
☐	☐	☐	☐	☐	☐	☐	☐	☐	☐	☐	☐

ACT Fidelity

Instructions for raters

Procedure

- The focus of this measure is on the therapist's behaviour.
- Therapists may not have the opportunity to demonstrate all behaviours captured by the ACT FM, especially in short sessions.
- Only score based on behaviours you have observed, not what you think the therapist would have achieved if they had further time available.
- A single therapist behaviour can be coded for all relevant items, not just the most suitable one.
- Before scoring the session, familiarise yourself with the measure and the items so that you can easily find an item when you see the clinician evidence it.
- Have specific examples in mind when scoring.
- Score the items at the end of the session not throughout, as ratings may change

Scoring

Give a rating for each item based on the behaviours you have heard or observed by circling the number next to each item. Items are rated as 0 if the behaviour did not occur, and from 1–3 if the behaviour did occur. Only assign a score higher than 0 if you hear or see examples of the behaviour. Higher scores are given for the behaviour occurring more consistently. Only give whole point answers, e.g. do not score 2.5. You will need to use your clinical judgment when scoring, bearing in mind the context of the therapy session and considering the function of the therapist behaviour.

Scoring

0 = This behaviour never occurred
1 = Therapist rarely enacts this behaviour
2 = Therapist sometimes enacts this behaviour
3 = Therapist consistently enacts this behaviour

Therapist Stance

	ACT consistent	Rating			
1.	Therapist chooses methods that are sensitive to the situation and context (i.e. in a flexible and responsive way rather than a 'one size fits all' approach).	0	1	2	3
2.	Therapist uses experiential methods/questions (i.e. helps the client to notice and use their own experience rather than thoughts about their experience).	0	1	2	3
3.	Therapist conveys that it is natural to experience painful or difficult thoughts and feelings when one is in circumstances such as those experienced by the client.	0	1	2	3
4.	Therapist demonstrates a willingness to sit with their own and the client's painful thoughts and feelings and the situations that give rise to these.	0	1	2	3

© Copyright University of Leeds 2021

Completed by ______ Date Day | | Month | | Year | | | |

Form continues on next page ►►

Prior to returning this form to CTRU you must make a copy of the form and any amendments for retention at site. CTRU, University of Leeds (please see Investigator Site File for full contact details).

**For office use only**	Computerised		Verified/Checked	
	Date	Initials	Date	Initials

Therapy Fidelity Assessment – ACT

ACT Fidelity *(Continued)*

Open Response Style

	ACT consistent	Rating			
5.	Therapist helps the client to notice thoughts as separate experiences from the events they describe.	0	1	2	3
6.	Therapist gives the client opportunities to notice how they interact with their thoughts and/or feelings (e.g. whether avoidant or open).	0	1	2	3
7.	Therapist encourages the client to “stay with” painful thoughts and feelings (in the service of their values).	0	1	2	3

Aware Response Style

	ACT consistent	Rating			
8.	Therapist uses present moment focus methods (e.g. mindfulness tasks, tracking, noticing, etc) to increase awareness of the moment, including thoughts and feelings.	0	1	2	3
9.	Therapist helps the client to notice the stimuli (thoughts, feelings, situations, etc.) that hook them away from the present moment.	0	1	2	3
10.	Therapist helps the client to experience that they are bigger than and/or separate from their psychological experiences.	0	1	2	3

Engaged Response Style

	ACT consistent	Rating			
11.	Therapist gives the client opportunities to notice workable and unworkable responses (e.g. whether their actions move them towards or away from their values).	0	1	2	3
12.	Therapist gives the client opportunities to clarify their own values (overarching life goals and qualities of action).	0	1	2	3
13.	Therapist helps the client to make plans and set goals likely to meet reinforcing consequences (i.e. shapes action that is consistent with their values).	0	1	2	3

Does the average rating score show fidelity to ACT? ☐ Yes ☐ No

© Copyright University of Leeds 2021

Completed by ________ Date Day | Month | Year

Last Page ■

Prior to returning this form to CTRU you must make a copy of the form and any amendments for retention at site. CTRU, University of Leeds (please see Investigator Site File for full contact details).

For office use only	Computerised		Verified/Checked	
	Date	Initials	Date	Initials
