# Supplementary material for: Randomised controlled trial of a psychotherapeutic intervention to improve quality of life and other outcomes in people who repeatedly self-harm: FReSH START study protocol
Source: Trials. 2024 Aug 26;25:564. doi: 10.1186/s13063-024-08369-2 (PMC11346196; doi:10.1186/s13063-024-08369-2)
Supplement: Supplementary file 5 — Additional file 5. FReSH START component fidelity checklist. [file 13063_2024_8369_MOESM5_ESM.pdf]

ISRCTN 73357210

FReSH START — Rethinking support for self-harm

FORM 37

FReSH START Fidelity Assessment

Participant ID number — Site Code | Trial No

Rater name

Number of sessions rated

Section A – Safety Components

- **Please rate the following components for session 1, 2 or 3**
- **You may rate other early sessions where components were not initially covered, if appropriate**

1. Therapist discusses with the client their prior experience of help and support.

   ☐ Yes ☐ No

2. Therapist spends time in the assessment exploring current, recent thoughts of SH and suicide and the likelihood of suicide in the next few days (i.e. prior to another session of therapy).

   ☐ Yes ☐ No

3. Therapist discusses with the client actions/strategies to keep the client safe during the week in-between sessions, and completes a safety plan

   ☐ Yes ☐ No

Section B – Non-specific Items from SPRS

- **For this section please only rate session 1**
- **Please refer to Rater's notes for scoring criteria (please tick appropriate score)**

4. WARMTH: Did the therapist convey warmth?

1	2	3	4	5	6	7
Not at all		Some		Quite a lot		Considerably

5. RAPPORT: How much rapport was there between therapist and client (i.e., how well did the therapist and client get along)?

1	2	3	4	5	6	7
Total absence of rapport		Some		Quite a lot		Considerable amount

6. EMPATHY: Was the therapist empathic toward the client (i.e. did she/he convey an intimate understanding of and sensitivity to the client's experiences and feelings)?

1	2	3	4	5	6	7
Not at all		Some		Quite a lot		Considerably

Completed by

Date — Day | Month | Year

Form continues on next page ►►

Prior to returning this form to CTRU you must make a copy of the form and any amendments for retention at site. CTRU, University of Leeds (please see Investigator Site File for full contact details).

For office use only	Computerised		Verified/Checked	
	Date	Initials	Date	Initials

Section C – FRESH START Components

7. Therapist discussed with client that SH has a purpose.

☐ Yes ☐ No

If yes, was the client accepting of this idea? ☐ Yes ☐ No

Was the therapist able to explore with the client its relevant functions? ☐ Yes ☐ No

Completed by ______________________ Date Day | Month | Year ____ *Last Page* ■

Prior to returning this form to CTRU you must make a copy of the form and any amendments for retention at site.
CTRU, University of Leeds (please see Investigator Site File for full contact details).

For office use only	*Computerised*		*Verified/Checked*	
	Date	*Initials*	*Date*	*Initials*
