# Supplementary material for: Randomised controlled trial of a psychotherapeutic intervention to improve quality of life and other outcomes in people who repeatedly self-harm: FReSH START study protocol
Source: Trials. 2024 Aug 26;25:564. doi: 10.1186/s13063-024-08369-2 (PMC11346196; doi:10.1186/s13063-024-08369-2)
Supplement: Supplementary file 6 — Additional file 6. 3 month Outcome Questionnaire. [file 13063_2024_8369_MOESM6_ESM.pdf]

Participant Questionnaire Booklet
• 3 Months

Rethinking support for self-harm

ISRCTN 73357210

To be completed by the researcher:

Participant Initials [| | |] Date of Birth Day [|] Month [|] Year [| | |] Participant ID Site Code [| | |] Trial No [| | | |]

Posting ☐ Initial ☐ Reminder 1

Questionnaire completed: ☐ By post ☐ By telephone ☐ Face-to-face

Thank you for agreeing to take part in the FReSH START study. This is a short questionnaire about the services you may have used in the past 3 months. **There are no right or wrong answers to any of the questions.**

Step 1. Please answer the questions to the best of your ability. We would be very grateful if you could complete all the questions.

Step 2. Please check that you have not missed any questions and return this questionnaire to us in the envelope provided. The postage has already been paid.

Please note: we will use this information for the purpose of the FReSH START study only. Your answers will remain **strictly confidential** and will not be shared outside of the trial team.

Thank you again for your valuable contribution to this research,

Elspeth Guthrie (Lead Researcher)

University of Leeds

This study is funded by the NIHR Programme Grants for Applied Research (PGfAR) RP-PG-1016-20005. The views expressed are those of the author(s) and not necessarily those of the NHS, the NIHR or the Department of Health and Social Care.

FUNDED BY NIHR | National Institute for Health Research

ctru University of Leeds

UNIVERSITY OF LEEDS

For office use only	Date Received	Date Entered (Date / Initials)

1. Residential care

In the **past three months**, have you had any residential stays (for example hostel, group home, crisis service)?

Yes ☐ No ☐

If yes, please give details in the table below

**Type of stay**	Length of stay in days

2. Primary and community care

Apart from hospital appointments, in the **past three months,** did you have contact with any other health professionals (e.g. your GP, a nurse) in the community?

Yes ☐ No ☐ (Go to Q3)

If yes, please give details in the table below.
If the type of professional isn't listed, please write this in 'Other'.

**Health professional**	Number of phone calls or online contact	Number of visits at practice	Number of visits at home
GP (Family doctor)			
Practice Nurse			
District nurse			
Physiotherapist			
Occupational therapist			
Drug and alcohol worker			
Mental Health worker including CPN, crisis, counsellor			
Social worker			
Help-line (e.g. Samaritans and 111)			
IAPT services			
Other services (e.g. addiction; charity / third sector), please specify …………………………………………………………			

3. Travel

During the **past three months**, how much money in total have you spent travelling to attend health or social care appointments, including planned (e.g. hospital and GP appointments) and unplanned visits (e.g. A&E)?

Please record any costs such as bus, taxis, train fares, petrol, car park fees etc.

£ …………………

Please check that you have completed all questions.

Thank you very much for your time
