# Supplementary material for: Randomised controlled trial of a psychotherapeutic intervention to improve quality of life and other outcomes in people who repeatedly self-harm: FReSH START study protocol
Source: Trials. 2024 Aug 26;25:564. doi: 10.1186/s13063-024-08369-2 (PMC11346196; doi:10.1186/s13063-024-08369-2)
Supplement: Supplementary file 9 — Additional file 9. 12 month Outcome Questionnaire. [file 13063_2024_8369_MOESM9_ESM.pdf]

Participant Questionnaire Booklet
• 12 Months

Rethinking support for self-harm

ISRCTN 73357210

To be completed by the researcher:

Participant Initials		Date of Birth	Day Month Year	Participant ID	Site Code Trial No
Posting	☐ Initial	☐ Reminder 1			
Questionnaire completed:	☐ By post	☐ By telephone	☐ Face-to-face		

Thank you for agreeing to take part in the FReSH START study. This questionnaire is divided into 5 sections, and **there are no right or wrong answers to any of the questions.**

Step 1. Please answer the questions to the best of your ability. We would be very grateful if you could complete all questions in each of the sections.

Step 2. Please check that you have not missed any questions or pages, and return this questionnaire to us in the envelope provided. The postage has already been paid.

Please note: we will use this information for the purpose of the FReSH START study only. Your answers will remain **strictly confidential** and will not be shared outside of the trial team.

Thank you again for your valuable contribution to this research,

Elspeth Guthrie (Lead Researcher)
University of Leeds

This study is funded by the NIHR Programme Grants for Applied Research (PGfAR) RP-PG-1016-20005. The views expressed are those of the author(s) and not necessarily those of the NHS, the NIHR or the Department of Health and Social Care.

FUNDED BY NIHR | National Institute for Health Research

ctru University of Leeds

UNIVERSITY OF LEEDS

For office use only	Date Received	Date Entered (Date / Initials)

This page is blank on purpose

Section 1: How are you feeling?

CORE-OM

Please write today's date .... / .... / .... (Day / Month / Year)

IMPORTANT - PLEASE READ THIS FIRST
This form has 34 statements about how you have been OVER THE LAST WEEK.
Please read each statement and think how often you felt that way last week.
Then tick the box which is closest to this.
Please use a dark pen (not pencil) and tick clearly within the boxes.

Over the last week

	Statement	Not at all	Only Occasionally	Sometimes	Often	Most or all the time	OFFICE USE ONLY
1	I have felt terribly alone and isolated	☐ 0	☐ 1	☐ 2	☐ 3	☐ 4	☐ F
2	I have felt tense, anxious or nervous	☐ 0	☐ 1	☐ 2	☐ 3	☐ 4	☐ P
3	I have felt I have someone to turn to for support when needed	☐ 4	☐ 3	☐ 2	☐ 1	☐ 0	☐ F
4	I have felt OK about myself	☐ 4	☐ 3	☐ 2	☐ 1	☐ 0	☐ W
5	I have felt totally lacking in energy and enthusiasm	☐ 0	☐ 1	☐ 2	☐ 3	☐ 4	☐ P
6	I have been physically violent to others	☐ 0	☐ 1	☐ 2	☐ 3	☐ 4	☐ R
7	I have felt able to cope when things go wrong	☐ 4	☐ 3	☐ 2	☐ 1	☐ 0	☐ F
8	I have been troubled by aches, pains or other physical problems	☐ 0	☐ 1	☐ 2	☐ 3	☐ 4	☐ P
9	I have thought of hurting myself	☐ 0	☐ 1	☐ 2	☐ 3	☐ 4	☐ R
10	Talking to people has felt too much for me	☐ 0	☐ 1	☐ 2	☐ 3	☐ 4	☐ F
11	Tension and anxiety have prevented me doing important things	☐ 0	☐ 1	☐ 2	☐ 3	☐ 4	☐ P
12	I have been happy with the things I have done	☐ 4	☐ 3	☐ 2	☐ 1	☐ 0	☐ F
13	I have been disturbed by unwanted thoughts and feelings	☐ 0	☐ 1	☐ 2	☐ 3	☐ 4	☐ P
14	I have felt like crying	☐ 0	☐ 1	☐ 2	☐ 3	☐ 4	☐ W

Please turn over

© CORE System Trust: http://www.coreims.co.uk/copyright.pdf
Supported by www.coreims.co.uk

Over the last week

	Not at all	Only Occasionally	Sometimes	Often	Most or all the time	OFFICE USE ONLY
15 I have felt panic or terror	☐ 0	☐ 1	☐ 2	☐ 3	☐ 4	☐ P
16 I made plans to end my life	☐ 0	☐ 1	☐ 2	☐ 3	☐ 4	☐ R
17 I have felt overwhelmed by my problems	☐ 0	☐ 1	☐ 2	☐ 3	☐ 4	☐ W
18 I have had difficulty getting to sleep or staying asleep	☐ 0	☐ 1	☐ 2	☐ 3	☐ 4	☐ P
19 I have felt warmth or affection for someone	☐ 4	☐ 3	☐ 2	☐ 1	☐ 0	☐ F
20 My problems have been impossible to put to one side	☐ 0	☐ 1	☐ 2	☐ 3	☐ 4	☐ P
21 I have been able to do most things I needed to	☐ 4	☐ 3	☐ 2	☐ 1	☐ 0	☐ F
22 I have threatened or intimidated another person	☐ 0	☐ 1	☐ 2	☐ 3	☐ 4	☐ R
23 I have felt despairing or hopeless	☐ 0	☐ 1	☐ 2	☐ 3	☐ 4	☐ P
24 I have thought it would be better if I were dead	☐ 0	☐ 1	☐ 2	☐ 3	☐ 4	☐ R
25 I have felt criticised by other people	☐ 0	☐ 1	☐ 2	☐ 3	☐ 4	☐ F
26 I have thought I have no friends	☐ 0	☐ 1	☐ 2	☐ 3	☐ 4	☐ F
27 I have felt unhappy	☐ 0	☐ 1	☐ 2	☐ 3	☐ 4	☐ P
28 Unwanted images or memories have been distressing me	☐ 0	☐ 1	☐ 2	☐ 3	☐ 4	☐ P
29 I have been irritable when with other people	☐ 0	☐ 1	☐ 2	☐ 3	☐ 4	☐ F
30 I have thought I am to blame for my problems and difficulties	☐ 0	☐ 1	☐ 2	☐ 3	☐ 4	☐ P
31 I have felt optimistic about my future	☐ 4	☐ 3	☐ 2	☐ 1	☐ 0	☐ W
32 I have achieved the things I wanted to	☐ 4	☐ 3	☐ 2	☐ 1	☐ 0	☐ F
33 I have felt humiliated or shamed by other people	☐ 0	☐ 1	☐ 2	☐ 3	☐ 4	☐ F
34 I have hurt myself physically or taken dangerous risks with my health	☐ 0	☐ 1	☐ 2	☐ 3	☐ 4	☐ R

© CORE System Trust: http://www.coreims.co.uk/copyright.pdf
Supported by www.coreims.co.uk

Section 2: Managing your day-to-day life

1. Employment

Please tick one box for the category that describes your employment status.

**Employment Status**	**Tick one category that best describes your employment now** *(please tick one box only)*
a. Employee, full time	☐
b. Employee, part time	☐
c. Self-employed	☐
d. Education or training	☐
f. Not in paid employment	☐
i. Other	☐

If you are not working, please go to Q2 – Help from other people

How many hours do you work in a typical week? ....

If you are working, what is your main job? ....

If you are currently working, have you needed time off work **because of any health problem** in the **past three months**?

Yes ☐ No ☐

If yes, please note how many days you've had off work .... days

People with health problems sometimes have to miss work because of their health problems. **Another possibility is that a person goes to work, but is unable to perform as well as they should because of health problems**. The following questions focus on these aspects.

Was your job performance worse than usual because of any health problem during the past three months?

No ☐ (Go to Q2 – Help from other people)

Yes ☐

On how many days **during the past three months did** you perform paid work, although you were bothered by health problems?

.... days (Please do not count any days on which you did not work at all because you called in sick.)

Please rate how well you performed on the days you went to work even though you were bothered by health problems. Please **tick one box** to rate how well you performed.
(1 indicates a considerably worse performance than usual and 5 that your work was not affected.)

1<br>My work performance was considerably worse than usual	2	3<br>My work performance was slightly worse than usual	4	5<br>My work performance was not affected
☐	☐	☐	☐	☐

2. Help from other people

In the **average week**, have you received assistance or help from anyone whilst at home?

Yes ☐ No ☐ (Go to Q3 – Money you've spent)

If yes, please indicate if you have received assistance or help with the following and indicate how many hours per day and days per week you received this help.

	Hours per day	Days per week
Personal care (e.g. washing/dressing/meal preparation)<br>Yes ☐ No ☐		
Help with domestic chores (e.g. cleaning)<br>Yes ☐ No ☐		
Other (...)<br>Yes ☐ No ☐		

Did the person who helped you have to take time off work in order to provide you with assistance or help?

Yes ☐ No ☐

If yes, how many days in an average week would they have to take off work? .... days

3. Medication

Have you been prescribed any medications by a doctor or other health professional **for your mental health** in the **past three months**? (e.g. pain killers, antidepressants, etc.).

Yes ☐ No ☐

If yes, please give details in the table below. (Please write in the names of the medications)

Name of medication	Dose	If dose unknown, number of tablets per day	When did you start this medication? (Write the date)	Are you still taking this medication?
				Yes ☐<br>No ☐
				Yes ☐<br>No ☐
				Yes ☐<br>No ☐

4. Self-harm

For this question please think about the **last 6 months**. In the past **6 months** how many times have you intentionally harmed yourself? (self-injury, self-poisoning both)?

Ways of harming yourself	Number of times self-harmed	Number of times attended hospital
Self-injury ☐<br>Self-poisoning ☐<br>Both ☐		

5. Money you've spent

In the **past three months**, have you spent any money **because of your self-harm**?

Yes ☐ No ☐

If yes, please give details in the table below.
Please write in what you spent your money on, and then how much it cost.

**Money spent due to self-harm**<br>E.g. you bought cream, patches, plasters, a self-help book or CD etc.	How much did you spend?

6. Residential care

In the **past three months**, have you had any residential stays (for example hostel, group home, crisis centre)?

Yes ☐ No ☐

If yes, please give details in the table below

**Type of stay**	Length of stay in days

7. Primary and community care

Apart from hospital appointments, in the **past three months,** did you have contact with any other health professionals (e.g. your GP, a nurse) in the community?

Yes ☐ No ☐ (Go to section 3)

If yes, please give details in the table below.
If the type of professional isn't listed, please write this in 'Other'.

**Health professional**	Number of phone calls or online contact	Number of visits at practice	Number of visits at home
GP (Family doctor)			
Practice Nurse			
District nurse			
Physiotherapist			
Occupational therapist			
Drug and alcohol worker			
Mental Health worker including CPN, crisis, counsellor			
Social worker			
Help-line (e.g. Samaritans and 111)			
IAPT services			
Other services (e.g. addiction; charity / third sector), please specify …………………………………………………………			

8. Travel

During the **past three months**, how much money in total have you spent travelling to attend health or social care appointments, including planned (e.g. hospital and GP appointments) and unplanned visits (e.g. A&E)?

Please record any costs such as bus, taxis, train fares, petrol, car park fees etc.

£ …………………

Section 3: Your thoughts and feelings

Beck Hopelessness Scale

BHS®

This questionnaire consists of 20 statements. Please read the statements carefully one by one. If the statement describes your attitude for the **past week, including today,** darken the circle with a 'T' indicating TRUE in the column next to the statement. If the statement does not describe your attitude, darken the circle with an 'F' indicating FALSE in the column next to this statement. **Please be sure to read each statement carefully.**

1. I look forward to the future with hope and enthusiasm. Ⓣ Ⓕ
2. I might as well give up because there is nothing I can do about making things better for myself. Ⓣ Ⓕ
3. When things are going badly, I am helped by knowing that they cannot stay that way forever. Ⓣ Ⓕ
4. I can't imagine what my life would be like in ten years. Ⓣ Ⓕ
5. I have enough time to accomplish the things I want to do. Ⓣ Ⓕ
6. In the future, I expect to succeed in what concerns me most. Ⓣ Ⓕ
7. My future seems dark to me. Ⓣ Ⓕ
8. I happen to be particularly lucky, and I expect to get more of the good things in life than the average person. Ⓣ Ⓕ
9. I just can't get the breaks, and there's no reason I will in the future. Ⓣ Ⓕ
10. My past experiences have prepared me well for the future. Ⓣ Ⓕ
11. All I can see ahead of me is unpleasantness rather than pleasantness. Ⓣ Ⓕ
12. I don't expect to get what I really want. Ⓣ Ⓕ
13. When I look ahead to the future, I expect that I will be happier than I am now. Ⓣ Ⓕ
14. Things just won't work out the way I want them to. Ⓣ Ⓕ
15. I have great faith in the future. Ⓣ Ⓕ
16. I never get what I want, so it's foolish to want anything. Ⓣ Ⓕ
17. It's very unlikely that I will get any real satisfaction in the future. Ⓣ Ⓕ
18. The future seems vague and uncertain to me. Ⓣ Ⓕ
19. I can look forward to more good times than bad times. Ⓣ Ⓕ
20. There's no use in really trying to get anything I want because I probably won't get it. Ⓣ Ⓕ

Pearson Executive Office 5601 Green Valley Drive Bloomington, MN 55437
800.627.7271 www.PsychCorp.com
Copyright © 1978 by Aaron T. Beck. All rights reserved.
Warning: No part of this publication may be reproduced or transmitted in any form or by any means, electronic or mechanical, including photocopy, recording, or any information storage and retrieval system, without permission in writing from the copyright owner.
Pearson, the **PSI logo, PsychCorp,** and **BHS** are trademarks in the U.S. and/or other countries of Pearson Education, Inc., or its affiliate(s).

PEARSON

PsychCorp

48 A B C D E 277613-4 65 Product Number 0154133620

Section 4: Your mental health

Patient Health Questionnaire-9 (PHQ- 9)

**Over the last 2 weeks, how often have you been bothered by any of the following problems?** *(Use "✔" to indicate your answer)*	**Not at all**	**Several days**	**More than half the days**	**Nearly every day**
**1.** Little interest or pleasure in doing things	0	1	2	3
**2.** Feeling down, depressed, or hopeless	0	1	2	3
**3.** Trouble falling or staying asleep, or sleeping too much	0	1	2	3
**4.** Feeling tired or having little energy	0	1	2	3
**5.** Poor appetite or overeating	0	1	2	3
**6.** Feeling bad about yourself — or that you are a failure or have let yourself or your family down	0	1	2	3
**7.** Trouble concentrating on things, such as reading the newspaper or watching television	0	1	2	3
**8.** Moving or speaking so slowly that other people could have noticed? Or the opposite — being so fidgety or restless that you have been moving around a lot more than usual	0	1	2	3
**9.** Thoughts that you would be better off dead or of hurting yourself in some way	0	1	2	3

FOR OFFICE CODING __*0*__ + ______ + ______ + ______

=Total Score: ______

If you checked off any problems, how difficult have these problems made it for you to do your work, take care of things at home, or get along with other people?

**Not difficult at all**	**Somewhat difficult**	**Very difficult**	**Extremely difficult**
☐	☐	☐	☐

Developed by Drs. Robert L. Spitzer, Janet B.W. Williams, Kurt Kroenke and colleagues, with an educational grant from Pfizer Inc. No permission required to reproduce, translate, display or distribute.

Section 5: Your relationships

The Social Connectedness Scale – Revised

Directions: Following are a number of statements that reflect various ways in which we view ourselves. Rate the degree to which you agree or disagree with each statement using the following scale (1 = Strongly Disagree and 6 = Strongly Agree). There is no right or wrong answer. Do not spend too much time with any one statement and do not leave any unanswered.

Strongly Disagree	Disagree	Mildly Disagree	Mildly Agree	Agree	Strongly Agree
**1**	**2**	**3**	**4**	**5**	**6**

		Strongly Disagree					Strongly Agree
1.	I feel comfortable in the presence of strangers.........	1	2	3	4	5	6
2.	I am in tune with the world......................................	1	2	3	4	5	6
*3.	Even among my friends, there is no sense of brother/sisterhood.......................................	1	2	3	4	5	6
4.	I fit in well in new situations....................................	1	2	3	4	5	6
5.	I feel close to people..	1	2	3	4	5	6
*6.	I feel disconnected from the world around me.........	1	2	3	4	5	6
*7.	Even around people I know, I don't feel that I really belong..	1	2	3	4	5	6
8.	I see people as friendly and approachable................	1	2	3	4	5	6
*9.	I feel like an outsider..	1	2	3	4	5	6
10.	I feel understood by the people I know....................	1	2	3	4	5	6
*11.	I feel distant from people..	1	2	3	4	5	6
12.	I am able to relate to my peers................................	1	2	3	4	5	6
*13.	I have little sense of togetherness with my peers.....	1	2	3	4	5	6
14.	I find myself actively involved in people's lives.....	1	2	3	4	5	6
*15.	I catch myself losing a sense of connectedness with society..	1	2	3	4	5	6
16.	I am able to connect with other people.....................	1	2	3	4	5	6
*17.	I see myself as a loner...	1	2	3	4	5	6
*18.	I don't feel related to most people............................	1	2	3	4	5	6
19.	My friends feel like family.......................................	1	2	3	4	5	6
*20.	I don't feel I participate with anyone or any group...	1	2	3	4	5	6
		Strongly Disagree					Strongly Agree

This is the end of the questionnaire.

Please check that you have completed all sections.

Thank you very much for your time

This page is blank on purpose

This page is blank on purpose
